# Real-world outcomes of bispecific antibody therapy after chimeric antigen receptor T-cell therapy in follicular lymphoma

**DOI:** 10.1038/s41408-026-01546-3

**Published:** 2026-06-18

**Authors:** John Sharp, Subodh Bhatta, Jennifer J. Huang, Colin J. Thomas, Omar Elghawy, Lindsey Fitzgerald, Daniel Reef, Andinet Teferra, Timothy J. Voorhees, Supriya Gupta, Natalie S. Grover, Stefan K. Barta, Mazyar Shadman, Narendranath Epperla

**Affiliations:** 1 James Comprehensive Cancer Center and Solove Research Institute, The Ohio State University, Columbus, OH USA; 2 Fred Hutchinson Cancer Center, University of Washington , Seattle, WA USA; 3 Abramson Cancer Center, University of Pennsylvania, Philadelphia, PA USA; 4 Huntsman Cancer Institute, University of Utah, Salt Lake City, UT USA; 5 Lineberger Comprehensive Cancer Center, University of North Carolina, Chapel Hill, NC USA; 6 Masonic Cancer Center, University of Minnesota, Minneapolis, MN USA

**Keywords:** B-cell lymphoma, Cancer immunotherapy

Dear Editor,

Follicular lymphoma (FL) is the second most common subtype of non-Hodgkin lymphoma, with approximately 14,000 new diagnoses annually in the United States. Although FL has historically been considered incurable, front-line therapy can induce durable remissions. Nevertheless, up to 50% of patients require multiple lines of treatment [1]. Importantly, the efficacy of therapy diminishes with successive lines [1], underscoring the importance of strategic sequencing of treatments in the management of FL.

Recently, the therapeutic landscape for relapsed/refractory (R/R) FL has expanded substantially with the introduction of novel immunotherapies, including chimeric antigen receptor T-cell (CAR-T) therapy and CD3 x CD20 bispecific antibody (BsAb) therapy [2]. Although both modalities leverage T-cell redirection to mediate antitumor activity, the optimal sequencing of these therapies in FL remains an important clinical question.

We previously described data collection for a real-world cohort of patients with R/R FL undergoing commercial CAR-T therapy with either axicabtagene ciloleucel or tisagenlecleucel [3]. Here, we describe outcomes among the subset of patients from this cohort who subsequently received commercial BsAb therapy for FL. Baseline clinical and disease characteristics were summarized descriptively, with medians and ranges for continuous variables and frequencies and percentages for categorical variables. Survival curves were generated using the Kaplan–Meier method. This study was approved by the institutional review boards of participating institutions, included a waiver of informed consent owing to its retrospective nature, and was conducted in accordance with the Declaration of Helsinki.

Among 136 patients who underwent commercial CAR-T therapy for R/R FL between 2021 and 2024, 18 (13%) subsequently received BsAb therapy. The median age at BsAb initiation was 60 years (range: 49–76). The median interval from CAR-T infusion to BsAb initiation was 18.6 months (range: 2.4–60.4 months), with 4 (22%) patients receiving BsAb ≤6 months following CAR-T infusion. The median number of prior lines of therapy (LOT) before BsAb initiation was 5 (range, 3–9), with 11 patients (61%) receiving BsAb as the immediate next line of therapy following CAR-T. Notably, 12 patients (67%) experienced progression of disease within 24 months of initial therapy (POD24). Among the 12 patients (67%) who underwent PET imaging prior to BsAb initiation, the median maximum SUV was 10.0 (range, 7.8–19.7). Importantly, among the 8 patients who underwent post–CAR-T biopsy, CD20 expression was confirmed in 4 (50%). BsAb therapies administered included mosunetuzumab in 15 patients (83%) and epcoritamab in 3 patients (17%) (Table 1).

**Table 1:** Baseline characteristics of patients with relapsed/refractory follicular lymphoma undergoing bispecific antibody therapy following chimeric antigen receptor T-cell therapy.

Characteristic	Total (*n* = 18)
Age in years—median (range)	60 (49–76)
Female sex—*n* (%)	5 (28%)
Comorbidities—*n* (%)	
Hypertension	5 (28%)
Diabetes mellitus	5 (28%)
Coronary artery disease	2 (11%)
COPD	1 (6%)
CKD	1 (6%)
POD24—*n* (%)	12 (67%)
Prior lines of therapy—median (range)	5 (3–9)
Prior autologous stem cell transplant—*n* (%)	2 (11%)
CAR-T LD regimen—*n* (%)	
Fludarabine/Cyclophosphamide	16 (89%)
Bendamustine	2 (11%)
CAR-T product utilized—*n* (%)	
Axicabtagene ciloleucel	14 (78%)
Tisagenlecleucel	4 (22%)
SUV max on PET prior to BsAb—median (range)^a^	10.0 (7.8–19.7)
CD20 expression confirmed post-CAR-T—*n* (%)^b^	4 (50%)
CAR-T to BsAb interval (months)—median (range)	18.6 (2.4–60.4)
BsAb initiation ≤ 6 months after CAR-T—*n* (%)	4 (22%)
Bispecific antibody used—*n* (%)	
Mosunetuzumab	15 (83%)
Epcoritamab	3 (17%)
Absolute neutrophil count (/µL)—median (range)	2480 (700–8600)
Absolute lymphocyte count (/µL)—median (range)	990 (200–2080)
Hemoglobin (g/dL)—median (range)	11.1 (8.2–14.5)
Platelets (K/µL)—median (range)	138 (5–345)
Lactate dehydrogenase (U/L)—median (range)	181 (93–569)

*BsAb* bispecific antibody therapy, *CAR-T* chimeric antigen receptor T-cell therapy, *CKD* chronic kidney disease, *COPD* chronic obstructive pulmonary disease, *CR* complete response, *LD* lymphodepletion, *PD* progressive disease, *PET* positron emission tomography, *POD24* progression of disease within 24 months of initial therapy, *PR* partial response, *SD* stable disease, *SUV* standardized uptake value.

^a^Pre-BsAb PET scan not obtained for 6 (33%) patients.

^b^Among the patients with post-CAR-T biopsy (*n* = 8), 4 had CD20 expression; post CAR-T biopsy was not performed in 10 patients.

Overall response rate (ORR) to BsAb therapy was 61% (95% CI: 36–83%) with a complete response rate (CRR) of 39% (95% CI: 17–64%, Table 2). Among patients who responded, median time to best response was 2.6 months (range: 1.8–9.4 months). By comparison, the ORR to prior CAR-T in this cohort was 72% (95% CI: 47–90%) with a CRR of 61% (95% CI: 36–83%, Table S1). Notably, among patients receiving BsAb ≤ 6 months from CAR-T infusion (*n* = 4), ORR was 50% (95% CI: 7–93%) with CRR of 0% compared to ORR of 64% (95% CI: 35–87%) and CRR of 50% (95% CI: 23–77%) among patients receiving BsAb > 6 months from CAR-T (*n* = 14, Table 2). Response rates according to POD24 status and prior LOT received are presented in Table S2 and Table S3, respectively.

**Table 2:** Responses to commercial CD3 x CD20 bispecific antibody therapy among patients with relapsed/refractory follicular lymphoma who had previously received commercial CD19-directed chimeric antigen receptor T-cell therapy by timing of bispecific antibody initiation.

Response rates to BsAb	Total (*n* = 18)	BsAb ≤ 6 months after CAR-T (*n* = 4, 22%)	BsAb > 6 months after CAR-T (*n* = 14, 78%)
Complete response	7 (39%)	0	7 (50%)
Partial response	4 (22%)	2 (50%)	2 (14%)
Stable disease	1 (6%)	0	1 (7%)
Progressive disease	6 (33%)	2 (50%)	4 (29%)
Overall response rate (95% CI)	61% (36–83%)	50% (7–93%)	64% (35–87%)
Complete response rate (95% CI)	39% (17–64%)	0% (0–60%)	50% (23–77%)

*BsAb* bispecific antibody therapy, *CAR-T* chimeric antigen receptor T-cell therapy.

At a median follow-up of 18.6 months, 11 (61%) patients progressed following BsAb. Median PFS was 6.0 months (95% CI: 4.6 months–not reached [NR], Fig. S1A). The 1-year PFS rate was 42% (95% CI: 24–73%). Seven (39%) patients died following BsAb initiation. Median OS was 23.6 months (95% CI: 13.4 months–NR, Fig. S1C). 1-year OS rate was 76% (95% CI: 58–100%). Notably, 6 of the 7 mortality events in this cohort were related to relapse. For patients starting BsAb ≤ 6 months after CAR-T infusion, median PFS and OS were 3.4 months (95% CI: 0.4 months–NR) and 16.2 months (95% CI: 1.4 months–NR), respectively, compared to 12.2 months (95% CI: 4.6 months–NR) and NR (95% CI: 18.4 months–NR) for those starting BsAb > 6 months after CAR-T (Fig. S1B, D). Univariable Cox regression analysis results for PFS and OS are presented in Table S4 and S5, respectively. Similarly, Kaplan-Meier curves for PFS and OS stratified by POD24 status and prior LOT are presented in Figs. S2 and S3, respectively.

Cytokine release syndrome (CRS) of any grade occurred in 9 (50%) patients, with all events limited to grade 1 (G1, *n* = 5, 28%) or G2 (*n* = 4, 22%) severity (Table S6). Two patients received tocilizumab, and 4 received steroids. No cases of immune effector cell-associated neurotoxicity syndrome (ICANS) were observed. Infections occurred in 5 patients (29%) during BsAb therapy, including 1 G2 and 4 G3 events, involving the respiratory tract (*n* = 3) and skin/soft tissue (*n* = 2). Incident G ≥ 3 cytopenias following BsAb initiation included neutropenia (G3 = 2, G4 = 1), anemia (G4 = 1), and thrombocytopenia (G3 = 1, G4 = 2).

Only 3 patients (17%) underwent planned hospitalization for BsAb administration (2 receiving epcoritamab, 1 receiving mosunetuzumab). However, 8 patients (44%) required unplanned hospitalization during BsAb therapy, including 4 for infections, 2 for CRS, 1 for an infusion reaction, and 1 for other reasons.

In this multicenter, retrospective cohort study of patients with R/R FL treated with BsAb therapy following prior CAR-T, we found that over half of patients achieved a response, although PFS was relatively short. Outcomes for patients initiating BsAb ≤ 6 months after CAR-T infusion were poor, though the small size of this cohort may obscure the true impact of BsAb timing on outcomes. Notable differences between this real-world cohort and patients enrolled in the clinical trials of epcoritamab [4] and mosunetuzumab [5] in FL merit consideration. Patients enrolled in these pivotal trials had received fewer prior LOT (median, 3 in both [4, 5]) compared with our cohort (median, 5). In addition, the proportion of patients with POD24 was lower in the trial populations (42% [4] and 52% [5]) than in our cohort (67%). These adverse features have been consistently associated with inferior response rates and shorter remission durations and likely contributed to the comparatively modest outcomes observed in this heavily pre-treated real-world population [1, 6]. Interestingly, we found no significant differences in our cohort between patients with and without POD24 or who had received ≤ or > 5 prior LOT.

The impact of prior CAR-T exposure on subsequent BsAb outcomes in R/R FL remains uncertain. Few patients enrolled in the clinical trials leading to regulatory approval of the BsAb products received prior CAR-T (*n* = 6 [4] and *n* = 3 [5]), limiting insight into this clinically relevant sequence. A short interval between CAR-T infusion and BsAb initiation has been associated with inferior outcomes following BsAb therapy in DLBCL [7], and we observed a similar association in FL. Acquired T-cell dysfunction following CAR-T [8] and inherent lymphoma-intrinsic treatment-resistance [7] may both contribute to this observation. Additional factors potentially influenced by prior CAR-T exposure include the biology of residual lymphoma subclones [9], alterations in the tumor microenvironment [10], and broader perturbations of host immune competence [11], all of which may modulate response to subsequent BsAb therapy. Additionally, as BsAbs are now approved in earlier LOT for FL [12], it may become more challenging to examine the effects of prior CAR-T on BsAb treatment outcomes. Despite the relatively modest PFS observed in this study, our findings demonstrate that clinically meaningful and durable responses to BsAb therapy are achievable in patients with FL previously treated with CAR-T.

The optimal sequencing of commercially available CAR-T and BsAb therapies in FL remains undefined, particularly among patients who may be eligible for either approach. In the absence of randomized data, a meta-analysis of clinical trials evaluating CAR-T and BsAb therapies in FL demonstrated higher response rates with CAR-T, although with a greater incidence of G ≥ 3 ICANS [13]. In contrast, G ≥ 3 infections were more frequently observed with BsAb therapy. Data from a meta-analysis in large B-cell lymphoma suggested that prior CAR-T exposure may attenuate responses to subsequent BsAb therapy [14]. Whether a similar relationship exists in FL is unknown; however, in our cohort, ORR and CRR were higher with CAR-T than with BsAb, consistent with these prior observations. Beyond efficacy and toxicity, logistical considerations are also relevant when selecting therapy for R/R FL. CAR-T therapy typically requires treatment at specialized centers and is associated with substantial time toxicity, whereas BsAb therapy may be more accessible and readily administered in the community setting [15]. Collectively, these factors underscore the complexity of treatment selection in R/R FL and suggest there may not be a single optimal approach to sequencing of therapy. Future studies should evaluate both clinical and patient-reported outcomes associated with specific sequencing strategies to better define optimal therapeutic approaches.

This analysis is limited by its retrospective design, modest sample size, and short duration of follow-up. Additionally, our dataset was limited to patients who received BsAb therapy following CAR-T progression, precluding the inclusion of a comparator cohort treated with non-BsAb approaches in the post–CAR-T relapse setting and limiting our ability to perform comparative outcome analyses.

Nevertheless, the current study adds meaningfully to the literature by characterizing a heavily pretreated population with R/R FL who achieved clinically significant, although relatively short-lived responses to BsAb therapy following CAR-T treatment. Notably, outcomes were poor among patients who initiated BsAb therapy within 6 months of CAR-T infusion. Further research is warranted to elucidate how the sequencing of these therapies influences lymphoma biology, clinical outcomes, and patient-reported experience, thereby informing shared decision-making and optimizing treatment strategies for patients with R/R FL.

## Supplementary information

Supplementary Appendix

## Data Availability

De-identified data will be made available on request to the corresponding author should the request meet the regulatory requirements per institutional IRB.
