# Supplementary material for: Real-world outcomes of bispecific antibody therapy after chimeric antigen receptor T-cell therapy in follicular lymphoma
Source: Blood Cancer J. 2026 Jun 18;16(1):96. doi: 10.1038/s41408-026-01546-3 (PMC13279774; doi:10.1038/s41408-026-01546-3)

**SUPPLEMENTAL APPENDIX**

**Table of Contents** 1

**Response to CAR-T in patients with relapsed/refractory follicular lymphoma who subsequently received BsAb:** Table S1 2

**Response to CAR-T in patients with relapsed/refractory follicular lymphoma who subsequently received BsAb by POD 24 status:** Table S2 3

**Response to CAR-T in patients with relapsed/refractory follicular lymphoma who subsequently received BsAb by lines of prior therapy:** Table S3 4

**Univariable Cox regression analysis for progression-free survival following BsAb in patients with relapsed/refractory follicular lymphoma after prior CAR-T:** Table S4 5

**Univariable Cox regression analysis for overall survival following BsAb in patients with relapsed/refractory follicular lymphoma after prior CAR-T:** Table S5 6

**Toxicities of BsAb in patients with relapsed/refractory follicular lymphoma after prior CAR-T:** Table S6 7

**PFS and OS for BsAb in patients with relapsed/refractory follicular lymphoma after prior CAR-T in overall cohort and by timing of BsAb initiation:** Figure S1 8

**PFS and OS for BsAb in patients with relapsed/refractory follicular lymphoma after prior CAR-T by POD24 status:** Figure S2 9

**PFS and OS for BsAb in patients with relapsed/refractory follicular lymphoma after prior CAR-T by lines of therapy:** Figure S3 10

**Table S1:** Responses to commercial CD19-directed CAR-T therapy among patients with relapsed/refractory follicular lymphoma who subsequently received commercial CD3 x CD20 bispecific antibody therapy

| Response to CAR-T | Total (n = 18) |
| --- | --- |
| Complete response | 11 (61%) |
| Partial response | 2 (11%) |
| Stable disease | 2 (11%) |
| Progressive disease | 3 (17%) |
| **Overall response rate (95% CI)** | 72% (47% - 90%) |
| **Complete response rate (95% CI)** | 61% (36% - 83%) |

Abbreviations: CAR-T=chimeric antigen receptor T-cell therapy

**Table S2:** Responses to commercial CD3 x CD20 bispecific antibody therapy among patients with relapsed/refractory follicular lymphoma who had previously received commercial CD19-directed chimeric antigen receptor T-cell therapy by POD24 status

| Response rates to BsAb | POD24 (n = 12, 67%) | No POD24 (n=6, 33%) |
| --- | --- | --- |
| Complete response | 5 (42%) | 2 (33%) |
| Partial response | 2 (17%) | 2 (33%) |
| Stable disease | 1 (8%) | 0 |
| Progressive disease | 4 (33%) | 2 (33%) |
| **Overall response rate (95% CI)** | 58% (28% - 85%) | 67% (22% - 96%) |
| **Complete response rate (95% CI)** | 42% (15% - 72%) | 33% (4% - 78%) |

Abbreviations: POD24=progression of disease within 24 months of initial treatment

**Table S3:** Responses to commercial CD3 x CD20 bispecific antibody therapy among patients with relapsed/refractory follicular lymphoma who had previously received commercial CD19-directed chimeric antigen receptor T-cell therapy by lines of prior therapy

| Response rates to BsAb | ≤5 (n = 11, 61%) | >5 (n=7, 39%) |
| --- | --- | --- |
| Complete response | 6 (55%) | 3 (43%) |
| Partial response | 1 (9%) | 1 (14%) |
| Stable disease | 2 (18%) | 1 (14%) |
| Progressive disease | 2 (18%) | 2 (29%) |
| **Overall response rate (95% CI)** | 64% (31% - 89%) | 57% (18% - 90%) |
| **Complete response rate (95% CI)** | 36% (11% - 69%) | 43% (10% - 82%) |

**Table S4:** Univariable Cox regression analysis for progression-free survival among patients with relapsed/refractory follicular lymphoma undergoing commercial CD3 x CD20 bispecific antibody therapy who had previously received commercial CD19-directed chimeric antigen receptor T-cell therapy by lines of prior therapy

| **Variable** | **N** | **HR (95% CI)** | **P-value** |
| --- | --- | --- | --- |
| **Number of Therapies before BsAb** | 18 |  |  |
| ≤5 | 11 | 1 (Ref.) |  |
| >5 | 7 | 0.60 (0.17–2.12) | 0.431 |
| **POD24** | 18 |  |  |
| No | 6 | 1 (Ref.) |  |
| Yes | 12 | 0.67 (0.19–2.36) | 0.537 |
| **Previous CAR-T Product** | 18 |  |  |
| Axicabtagene ciloleucel | 14 | 1 (Ref.) |  |
| Tisagenlecleucel | 5 | 3.32 (0.92–12.0) | 0.066 |
| **Bispecific Antibody** | 18 |  |  |
| Mosunetuzumab | 15 | 1 (Ref.) |  |
| Epcoritamab | 3 | 0.41 (0.05 – 3.20) | 0.394 |

Abbreviations: BsAb=bispecific antibody, CAR-T=chimeric antigen receptor T-cell, CI = Confidence Interval, HR = Hazard Ratio, POD24=progression of disease within 24 months of initial therapy

**Table S5:** Univariable Cox regression analysis for overall survival among patients with relapsed/refractory follicular lymphoma undergoing commercial CD3 x CD20 bispecific antibody therapy who had previously received commercial CD19-directed chimeric antigen receptor T-cell therapy by lines of prior therapy

| **Variable** | **N** | **HR (95% CI)** | **P-value** |
| --- | --- | --- | --- |
| **Number of Therapies before BsAb** | 18 |  |  |
| ≤5 | 11 | 1 (Ref.) |  |
| >5 | 7 | 0.50 (0.10–2.57) | 0.404 |
| **POD24** | 18 |  |  |
| No | 6 | 1 (Ref.) |  |
| Yes | 12 | 0.76 (0.14–4.25) | 0.757 |
| **Previous CAR-T Product** | 18 |  |  |
| Axicabtagene ciloleucel | 14 | 1 (Ref.) |  |
| Tisagenlecleucel | 5 | 3.38 (0.75–15.3) | 0.114 |
| **Bispecific Antibody** | 18 |  |  |
| Mosunetuzumab | 15 | 1 (Ref.) |  |
| Epcoritamab | 3 | 1.60 (0.18 – 14.4) | 0.677 |

Abbreviations: BsAb=bispecific antibody, CAR-T=chimeric antigen receptor T-cell, CI = Confidence Interval, HR = Hazard Ratio, POD24=progression of disease within 24 months of initial therapy

**Table S6:** Toxicities among patients undergoing commercial CD3 x CD20 bispecific antibody therapy for relapsed/refractory follicular lymphoma who previously received commercial CD19-directed chimeric antigen receptor T-cell therapy

| Toxicity | Total  (n = 18) |
| --- | --- |
| **CRS grade** – n (%)  Any grade  Grade ≥3 | 9 (50%)  0 |
| **ICANS grade** – n (%)  Any grade  Grade ≥3 | 0  0 |
| **Infection** – n (%)  Any grade  Grade ≥3 | 5 (29%)  4 (22%) |
| **Neutropenia** – n (%)  Any grade  Grade ≥3 | 3 (17%)  3 (17%) |
| **Anemia** – n (%)  Any grade  Grade ≥3 | 3 (17%)  1 (6%) |
| **Thrombocytopenia** – n (%)  Any grade  Grade ≥3 | 3 (17%)  3 (17%) |

Abbreviations: CRS=cytokine release syndrome, ICANS=immune effector cell neurotoxicity syndrome

**Figure S1:** Outcomes among patients with relapsed/refractory follicular lymphoma undergoing bispecific antibody therapy after previously receiving chimeric antigen receptor T-cell therapy A) Progression-free survival in overall cohort B) progression-free survival by timing of bispecific antibody initiation in relation to chimeric antigen receptor T-cell infusion C) overall survival in overall cohort D) overall survival by timing of bispecific antibody initiation in relation to chimeric antigen receptor T-cell infusion


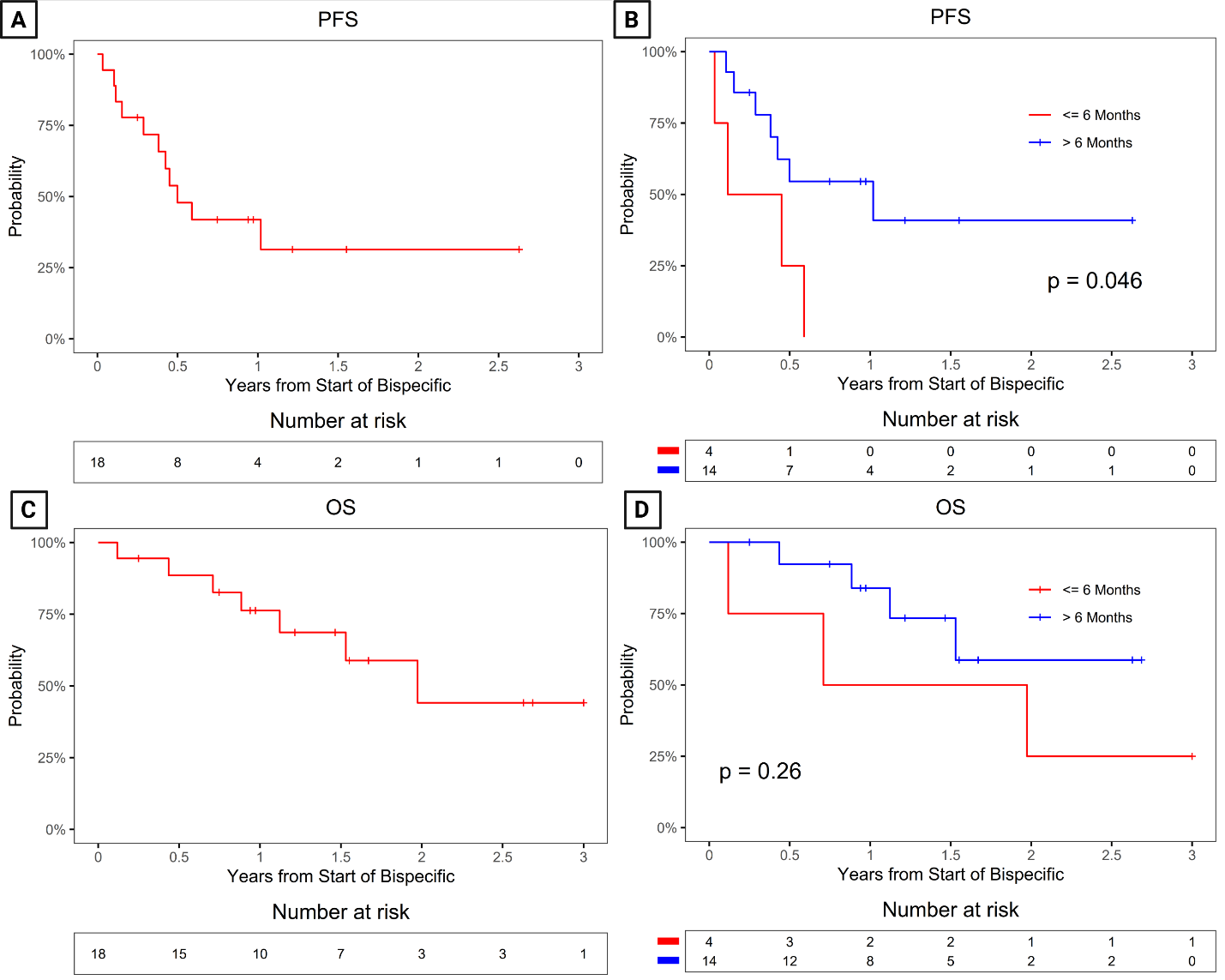


Abbreviations: PFS=progression-free survival, OS=overall survival

**Figure S2:** Outcomes among patients with relapsed/refractory follicular lymphoma undergoing bispecific antibody therapy after previously receiving chimeric antigen receptor T-cell therapy according to POD24 status A) progression-free survival B) overall survival.


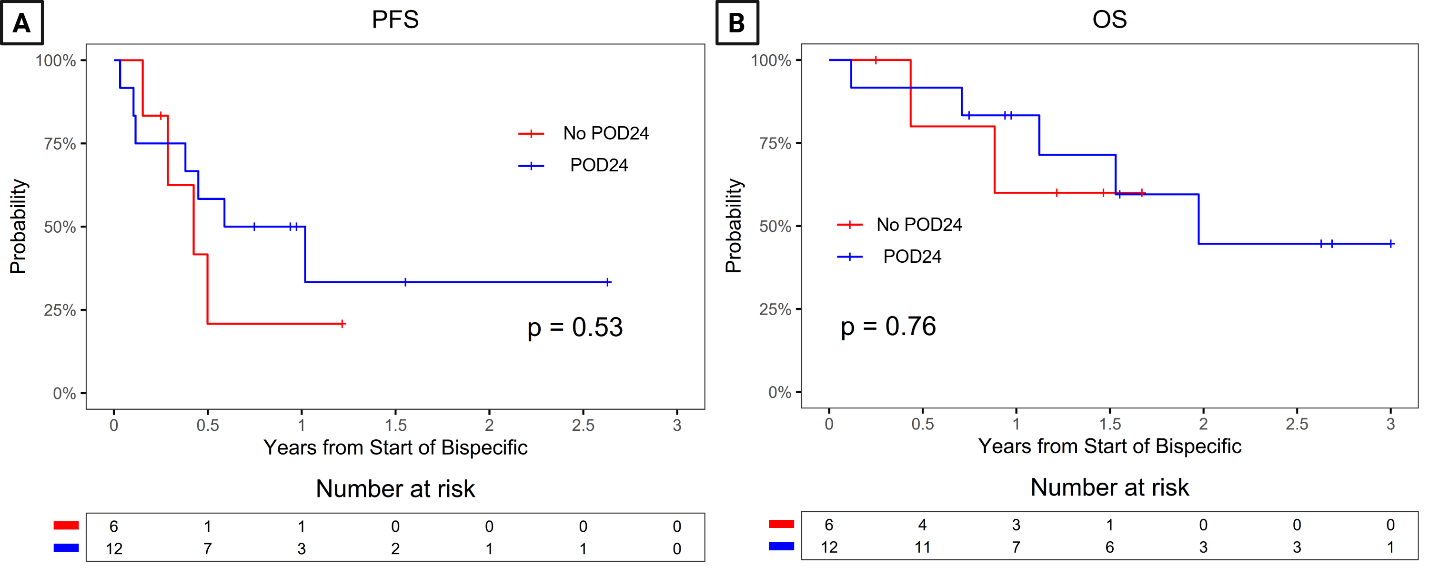


**Figure S3:** Outcomes among patients with relapsed/refractory follicular lymphoma undergoing bispecific antibody therapy after previously receiving chimeric antigen receptor T-cell therapy according to prior lines of therapy received A) progression-free survival B) overall survival.


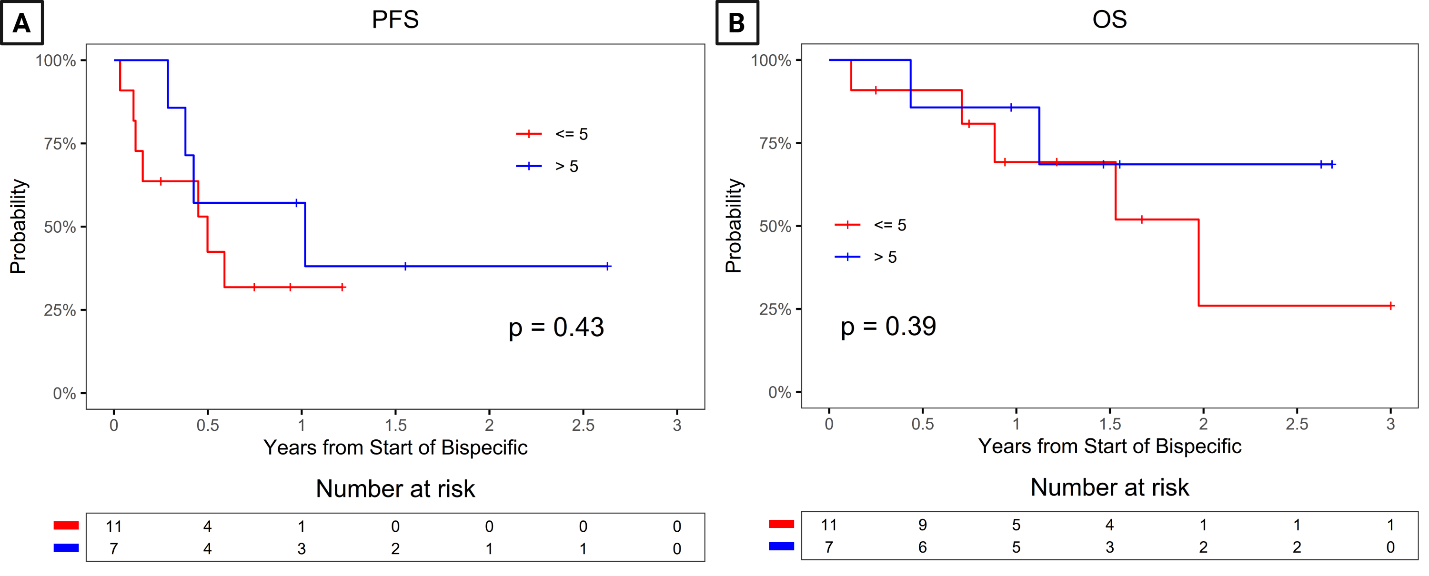

Supplement: Supplementary file 1 — Supplementary Appendix [file 41408_2026_1546_MOESM1_ESM.docx]
